# Editorial: Application of plant secondary metabolites to pain neuromodulation, volume II

**DOI:** 10.3389/fphar.2022.1013063

**Published:** 2022-09-26

**Authors:** Rajeev K. Singla, Adriana Gibara Guimarães, Gokhan Zengin

**Affiliations:** ^1^ Institutes for Systems Genetics, Frontiers Science Center for Disease-Related Molecular Network, West China Hospital, Sichuan University, Chengdu, China; ^2^ School of Pharmaceutical Sciences, Lovely Professional University, Phagwara, India; ^3^ Federal University of Sergipe, São Cristóvão, Brazil; ^4^ Selçuk University, Konya, Turkey

**Keywords:** pain, nociception, natural products, secondary metabolites, phytochemicals, medicinal plants, polyphenol

Pain is an uncomfortable condition that is clinically associated with many diseases such as cancer/tumor, metabolic disorders such as diabetes, neurological diseases such as epilepsy and chronic infectious diseases, and functional disorders (Li et al., 2019; Yang, 2019; Singla et al., 2021a; Singla et al., 2021b). The neuromodulation approach has used electrical interfaces to modulate neuronal activity and has proven effective in treating various neurological disorders, including chronic pain (James et al., 2018). It has been adopted and accepted as an alternative techniques because of high attrition rates, costs as well as regulatory conditions in case of pharmacological agents (James et al., 2018). But in order to ascertain the aesthetic and appropriate usage of neurostimulation technique, organizations like “The International Neuromodulation Society (INS)” have framed the practice guidelines (Deer et al., 2014a; Deer et al., 2014b). Even for the cancer pain management, “The Indian Society for Study of Pain (ISSP)” have also prepared guidelines to cover palliative care aspects (Thota et al., 2020). As an alternative to both the synthetic pharmacological agents as well as neurostimulaory techniques, natural products have had a lot of translational potential to reach the bedside (Singla et al., 2021b; Dangar and Patel, 2021; Swarnkar et al., 2021; Rauf et al., 2022). Infact, nature has gifted us with lot of such type of molecules like capsaicin, resinferatoxin, morphine, lipoxin A4, cannabidiol, etc that have a strong potential in pain alleviation (Jin et al., 2020; Singla et al., 2020; Singla et al., 2021b). Nanotechnological approaches further augment the pharmacological properties of the therapeutic agents (Yetisgin et al., 2020; Annaso et al., 2022). Mishra and the team have discussed the clinical translational potential of gold nanoparticles as an effective neuromedicines (Mishra et al., 2022). This Research Topic was thus planned to cover various aspects related to the plant metabolites for pain neuromodulation to gather further insights in this direction.

Bone cancer pain is unique in that it shares salient characteristics of neuropathic, nociceptive, and inflammatory pain (de Clauser et al., 2020). Metformin is a semi-synthetic analogue of the natural product present there in *Galega officinalis* L. (Hardie, 2022). Qian et al. in their research article entitled “Metformin Attenuates Bone Cancer Pain by Reducing TRPV1 and ASIC3 Expression” studied the effects of metformin in bone cancer pain model of rats and compared with the results of capsazepine, a “transient receptor potential cation channel subfamily V member 1 (TRPV1)” inhibitor and amiloride, an “Acid-sensitive ion channel 3 (ASIC3)” antagonist. They have observed that metformin has a capability to increase the paw withdraw threshold, as well as able to reduce TRPV1 expression in L4-6 dorsal root ganglions (DRG) and L4-6 spinal dorsal horn (SDH), while reducing expression of ASIC3 in L4-6 SDH. This suggested the potential of metformin in alleviating bone cancer pain.

Chinese herbal medicine like *Reynoutria multiflora* Thunb (*Polygonum multiflorum* Thunb.) is well known for multiple therapeutic properties like Cerebral Ischemic Reperfusion Injury (Huang P. et al., 2022), neurodegenerative diseases, and inflammation (Feng and Bounda, 2015). It is officially listed in Chinese Pharmacopoeeia and popularly known as “He shou wu” in China Mainland (Feng and Bounda, 2015; Li et al., 2017). Bai et al. in their research article entitled “Transformation of Stilbene Glucosides From Reynoutria multiflora During Processing” studied the transformation of stilbene glucosides and observed the changes bewteen raw from and the processed form. They have developed a simple and effective protocol using UHPLC-Q-Exactive plus orbitrap MS/MS. They have also observed that the number of transformed compounds are processing time dependent too.

Neuropathic pain and neuroinflammation are often linked with the nerve injuries like scientic nerve injury (Myers et al., 2006; Ellis and Bennett, 2013; Mahmoud et al.). Literature suggested that *Potamogeton perfoliatus* L. inhibiting 5-lipoxygenase and cyclooxygenase-2 enzymes, and thus possessing potential anti-inflammatory and analgesic properties (Rezq et al., 2021). Mahmoud et al. in their research article entitled “Potamogeton perfoliatus L. Extract Attenuates Neuroinflammation and Neuropathic Pain in Sciatic Nerve Chronic Constriction Injury-Induced Peripheral Neuropathy in Rats” has studied the hydroalcholic extract (whole plant) of *Potamogeton perfoliatus* L. on “sciatic nerve chronic constriction injury rat model”. They have noticed that the extract was having multitargeted potential and targeting various enzymes/receptors as well as pathways while modulating and attenuating the neuroinflammation and neuropathic pain in the tested animals.

Proanthocyanidin extract was reported to have anti-hyperalgesic and anti-nociceptive potentials when tested in rat model with neuropathic pain (Kaur et al., 2016). El-Shitany and Eid further confirmed the protective effects of proanthocyanidin against cisplatin-induced liver damage through alleviation of inflammation and modulation of NF-κβ/TLR-4 pathway (El-Shitany and Eid, 2017). Fan et al. in their research article “Proanthocyanidins Inhibit the Transmission of Spinal Pain Information Through a Presynaptic Mechanism in a Mouse Inflammatory Pain Model” had found that proanthocyanidin has a potent inflammatory pain relieving ability when studied in mice with Complete Freund’s Adjuvant injection. The possible mechanism for this effect is the modulation of PI3K/Akt/mTOR pathway in DRGs.

Diabetic patients are commonly facing complication like diabetic neuropathic pain (Xie et al., 2022). There are various mechnisms channeling diabetic neuropathic pain like WNT-mediated TRPV1 activation (Xie et al., 2022), “thioredoxin-interacting protein (TXNIP)-NOD-like receptor protein 3 (NLRP3)-N-methyl-D-aspartic acid receptor 2B (NR2B) pathway” (Wang J.-W. et al., 2022), P2Y_14_ receptor (Wu et al., 2022), ASK1-MKK3-p38 pathway (Wang F. et al., 2022), NLRP3 (Zhang et al., 2022), P2X7R expression (Hu et al., 2022), along with many others. Omar et al. in their systematic review article entitled “Tannins in the Treatment of Diabetic Neuropathic Pain: Research Progress and Future Challenges” had systematically analyzed the research focused on the tannins for their alleviating effects on diabetic neuropathic symptoms. They concluded that the effects most probably is through the hypoglycaemic effect of these phytochemical tannins.

Alzheimer’s disease (AD) is often associated as co-morbid with chronic pain (Cao et al., 2019). Bhat et al. in their review article entitled “Natural Therapeutics in Aid of Treating Alzheimer’s Disease: A Green Gateway Toward Ending Quest for Treating Neurological Disorders” has analysed the literature encompassing natural products having anti-alzheimer’s potential. In the article, they have discussed various pathologies associated with Alzheimer’s disease like that related to cholinergic, tau protein, amyloid-β, neuroinflammation, and oxidative stress. They had further discussed various natural Anti-alzheimer’s agents like ellagic acids as having anti-amyloidogenic property, punicalagin as β-secretase inhibitor, curcumin having tau hypophosphorylation effect, along with many other Anti-alzheimer’s agents. They had covered literature for around 24 medicinal herbs and 22 phytochemicals having potential to manage Alzheimer’s disease.

Orofacial pain primarily affects the head, face, and neck areas and is generally associated with inflammation (Romero-Reyes and Uyanik, 2014). Natural products, especially terpenes are effective in modulating orofacial nociception (Silva et al., 2016; Oliveira et al., 2020). Myrtenol in complex with β-cyclodextrin has been able to elicit anti-nociceptive behavior and cognitive enhancement in a chronic musculoskeletal pain model (Heimfarth et al., 2020). Oliveira et al. in their brief research report article entitled “Myrtenol Reduces Orofacial Nociception and Inflammation in Mice Through p38-MAPK and Cytokine Inhibition” have evaluated the therapeutic potential of myrtenol in reducing orofacial pain and inflammation in formalin-induced pain model of male Swiss mice. They have further demonstrated possible mechanisms as modulation of IL-1β levels in the trigeminal pathway as well as p38-MAPK modulation in trigeminal ganglia.

Consuming of various forms of ginger (*Zingiber officinale* Roscoe) such as ginger extract, ginger essential oils, etc have tremendous antineuropathic effects including thermal and cold hyperalgesia (Shen et al., 2022a; Shen et al., 2022b). Shen et al. in their original research article entitled “Gingerol-Enriched Ginger Supplementation Mitigates Neuropathic Pain via Mitigating Intestinal Permeability and Neuroinflammation: Gut-Brain Connection” have presented a noteworthy role of gut-brain axis in mitigation of the neuropathic pain that were validated by the *in vivo* experiments on male rats.

Chinese herbs and the traditional Chinese medicines (TCM) are known for their role in modulating of pain and inflammation, and most of them are now experimentally validated (Chen and Zhang, 2014; Du et al., 2016). One such formulation is Xiongshao Zhitong Recipe (XZR, a combination of eight botanical drugs), which is traditionally being indicated for migraine, but mechanisms behind it were not clear (Yang et al., 2022). Keeping this in mind, Yang et al. in their original research article entitled “Xiongshao Zhitong Recipe Attenuates Nitroglycerin-Induced Migraine-Like Behaviors via the Inhibition of Inflammation Mediated by Nitric Oxide Synthase” have done the phytochemical characterization of this TCM using UHPLC-LTQ-Orbitrap MS assay, and validated the antimigraine activity of the aqueous extract obtained from XZR using their own developed rat model with nitroglycerin induced migraine. They have observed that the nitric oxide synthase suppression and NF-κB signaling pathway activation are the possible mechanisms behind the anti-inflammatory activity of XZR.

Previous studies validated the alleviating role of terpenes in neuropathic pain (Bortalanza et al., 2002; Borgonetti et al., 2020; Bilbrey et al., 2021). Earlier studies have indicated that pristimerin is having anti-inflammatory activity and possibly having it by modulation various pathways like NF-κB pathway (Huang D. et al., 2022), PI3K/Akt signalling (Xue et al., 2021), NLRP3 (Zhao et al., 2020), etc. Al-Romaiyan and Masocha in their original research article entitled “Pristimerin, a triterpene that inhibits monoacylglycerol lipase activity, prevents the development of paclitaxel-induced allodynia in mice” have observed that pristimerin is having potent and dose-dependent monoacylglycerol inhibitor when compared with JZL-195, betulinic acid, cucurbitacin B, and euphol. Upregulation of Nrf2 gene expression was also observed.

This Research Topic, thus covered 1 brief research report, 7 original research, 1 review, and 1 systematic review article. As on 4^th^ August, 2022, there were cumulative 10,364 views of the 10 articles published in this Research Topic, with cumulative 2,185 downloads as per Frontiers record. We are highly thankful to all the authors for contributing their scholarly work in our Research Topic and we are indeed grateful to all the reviewers who had spared time from their tight schedule and supported us in processing of these manuscripts. This Research Topic is providing a good overview about the natural products having potential against various types of neuropathic pain and neuroinflammation like bone cancer pain, orofacial pain, diabetic neuropathic pain, spinal pain, etc. It is thus very important to do further translational studies to assess the clinical level application of these natural products.
